# Distribution of PEG-coated hollow polyelectrolyte microcapsules after introduction into the circulatory system and muscles of zebrafish

**DOI:** 10.1242/bio.030015

**Published:** 2018-01-15

**Authors:** Ekaterina Borvinskaya, Anton Gurkov, Ekaterina Shchapova, Boris Baduev, Igor Meglinski, Maxim Timofeyev

**Affiliations:** 1 Institute of Biology at Irkutsk State University, Irkutsk 664003, Russia; 2 Institute of Biology at Karelian Research Centre of Russian Academy of Sciences, Petrozavodsk 185035, Russia; 3 Baikal Research Centre, Irkutsk 664003, Russia; 4 University of Oulu, Optoelectronics and Measurement Techniques Laboratory, Oulu 90570, Finland

**Keywords:** Fluorescent probes, *In vivo* sensing, Layer-by-layer, Microencapsulated biomarkers

## Abstract

The use of polyelectrolyte multilayer microcapsules as carriers for fluorescent molecular probes is a prospective technique for monitoring the physiological characteristics of animal vasculature and interstitial environment *in vivo*. Polyelectrolyte microcapsules have many features that favor their use as implantable carriers of optical sensors, but little information is available on their interactions with complex living tissues, distribution or residence time following different routes of administration in the body of vertebrates. Using the common fish model, the zebrafish *Danio rerio*, we studied *in vivo* the distribution of non-biodegradable microcapsules covered with polyethylene glycol (PEG) over time in the adults and evaluated potential side effects of their delivery into the fish bloodstream and muscles. Fluorescent microcapsules administered into the bloodstream and interstitially (in concentrations that were sufficient for visualization and spectral signal recording) both showed negligible acute toxicity to the fishes during three weeks of observation. The distribution pattern of microcapsules delivered into the bloodstream was stable for at least one week, with microcapsules prevalent in capillaries-rich organs. However, after intramuscular injection, the phagocytosis of the microcapsules by immune cells was manifested, indicating considerable immunogenicity of the microcapsules despite PEG coverage. The long-term negative effects of chronic inflammation were also investigated in fish muscles by histological analysis.

## Abstract (precis)

**Summary:** With remote physiological sensing in mind, the study explores the fate of fluorescent microcapsules with elastic shell after injection in bloodstream and muscles of adult zebrafish.

## INTRODUCTION

Microencapsulation is a collection of techniques with great potential for application in biosciences. One of the most promising methods for microencapsulation is the template-assisted approach via layer-by-layer (LbL) assembly of oppositely charged polyelectrolytes (Decher, 1997; De Geest et al., 2006; Cui et al., 2014; Liu and Picart, 2016; Vilela et al., 2017). This technique allows fast and easy preparation of hollow microcapsules with a hydrophilic semipermeable polymeric wall enclosing some functional compound (Donath et al., 1998; Antipov et al., 2003; Volodkin et al., 2004; Gaponik et al., 2003; De Geest et al., 2006). The LbL-assembled microcapsules have two general types of potential applications in medicine and biological research on whole organisms. (1) Delivery of drugs and vaccines to target organs/tissues; this requires some technological solutions for control of the wall opening of capsules (De Geest et al., 2007, 2012; Esser-Kahn et al., 2011; Skirtach et al., 2011; Dierendonck et al., 2012; Feng et al., 2014; Gao et al., 2016). (2) Sensing of physiological parameters *in vivo* (Ruckh and Clark, 2014; Cui et al., 2014; Gurkov et al., 2016; Borvinskaya et al., 2017). This employment is based on the properties of semipermeability and stability (at least on certain time scales) of some types of polymeric shells.

Although drug delivery with polyelectrolyte microcapsules (PMs) is currently the primary vector in the development of PM technology (Antipina et al., 2014), monitoring of different physiological characteristics *in vivo* also offers significant prospects. Immobilization of optical molecular probes into PMs is an emerging technique in implantable sensor design for several reasons. The fluorescent molecular probes sensitive to certain metabolites, ions (including H^+^, Na^+^ and Ca^2+^) and oxygen radicals (Johnson and Spence, 2010) can have considerable toxicity to the studied organism (Alford et al., 2009), and they dissolve in the whole volume of internal fluids, which complicates acquisition of the fluorescent signal. Packing of dyes into semipermeable shells of PMs prevents their bioavailability and decreases toxicity; thus, the probes are resistant to biodegradation and the fluorescent signal is intensified. When applied *in vivo*, covering the PMs with a polyethylene glycol-containing (PEG) polymer can significantly decrease their aggregation and sticking to walls of blood capillaries, which minimizes the toxicity from the PMs, and mimic PMs from phagocytes at least during several hours (Wattendorf et al., 2008; Knop et al., 2010; Sadovoy et al., 2012). Moreover, flexible and thin walls of micron-sized PMs allow flexibility in small capillaries, similar to that of blood cells, for distribution in the circulatory system.

Although the cytotoxicity of PMs has received considerable attention (Wattendorf et al., 2008; De Koker et al., 2007; Minaeva et al., 2017), their toxicity at the organism level, interaction with complex living tissues and fate in organisms depending on the route of administration remain poorly reported (Zhao et al., 2007; De Koker et al., 2007, 2010; Sadovoy et al., 2012; Yi et al., 2014; Shao et al., 2015; Voronin et al., 2017). Importantly, the most studies report on distribution of microcapsules in certain organs or regions of the body (mostly tumors), and lack analysis of microcapsule behavior on the organism level. For the prospective applications of PMs in diverse areas, the methodology of delivery and visualization (for optical sensing) of PMs in different parts and organs of vertebrate organisms must be developed and optimized.

This article provides the first practical steps toward a consistent technique for implanting PMs with the PEG-containing coverage (PMs-PEG) into adult zebrafish *Danio rerio* (Hamilton 1822). This species is a common model for investigation of stress physiology and metabolic disorders in vertebrates, and also is a traditional organism in aquatic ecotoxicology (Pereira et al., 2016; Nguyen et al., 2013). Because the zebrafish has a small and translucent body (transparent strains are also developed), this fish is more suitable as a vertebrate subject than adult rodents to study the distribution of fluorescent PMs. Because our primary focus was the further application of PMs-PEG for physiological measurements, stable non-biodegradable materials, such as poly(allylamine hydrochloride) (PAH) and poly(sodium 4-styrenesulfonate) (PSS), were selected to form the semipermeable wall of the PMs. In this study, we administered PMs-PEG to adult *D. rerio* to investigate their distribution in tissues, stability, toxicity and general side effects, as well as to practice their visualization inside the organism using different strategies of delivery. We examined the intramuscular route of injection and introduction into the bloodstream and recorded short-term and long-term *in vivo* effects of PMs-PEG on the zebrafish organism.

## RESULTS

### Organism-level toxicity of PMs-PEG

In an earlier study, the toxicity of PMs-PEG to developing embryos of *D. rerio* was low (Sadovoy et al., 2012). Based on our data, the use of PMs-PEG in adult *D. rerio* also resulted in low acute side effects. After intramuscular injection with different types of PMs-PEG, nearly all fishes recovered fully and swam actively in their holding tanks (<2% mortality, *n*=101; Table S1). We did not observe delayed effects of the injections on survival in the control group after 7 and 14 days (*n*=50 and 5, respectively).

After injection into the kidney (for implantation of PMs-PEG into the bloodstream), the most deaths (10-15%) were registered during the first few hours after the injection in both experimental (*n*=119) and control groups (*n*=37) (Table S1). This mortality was most likely associated with the trauma and stress caused by the procedure. In general, fishes treated with PMs-PEG of an average size of 5.1 µm had lower survival than fishes injected with microcapsules approximately 2 μm in diameter, which had the best survival. Among fishes that were injected with PMs-PEG containing the dye FITC-BSA, the highest mortality during the experiment was among fishes that received the largest concentration of the microcapsules (approximately 4×10^6^ µl), compared with fishes injected with lower concentrations.

Based on the results, mortality was not significantly affected by either route of administration of PMs-PEG, and therefore, these PMs-PEG could be used for implanting of microcapsules in experimental procedures.

### Distribution of PMs-PEG in the bloodstream

**Fig. 1.**
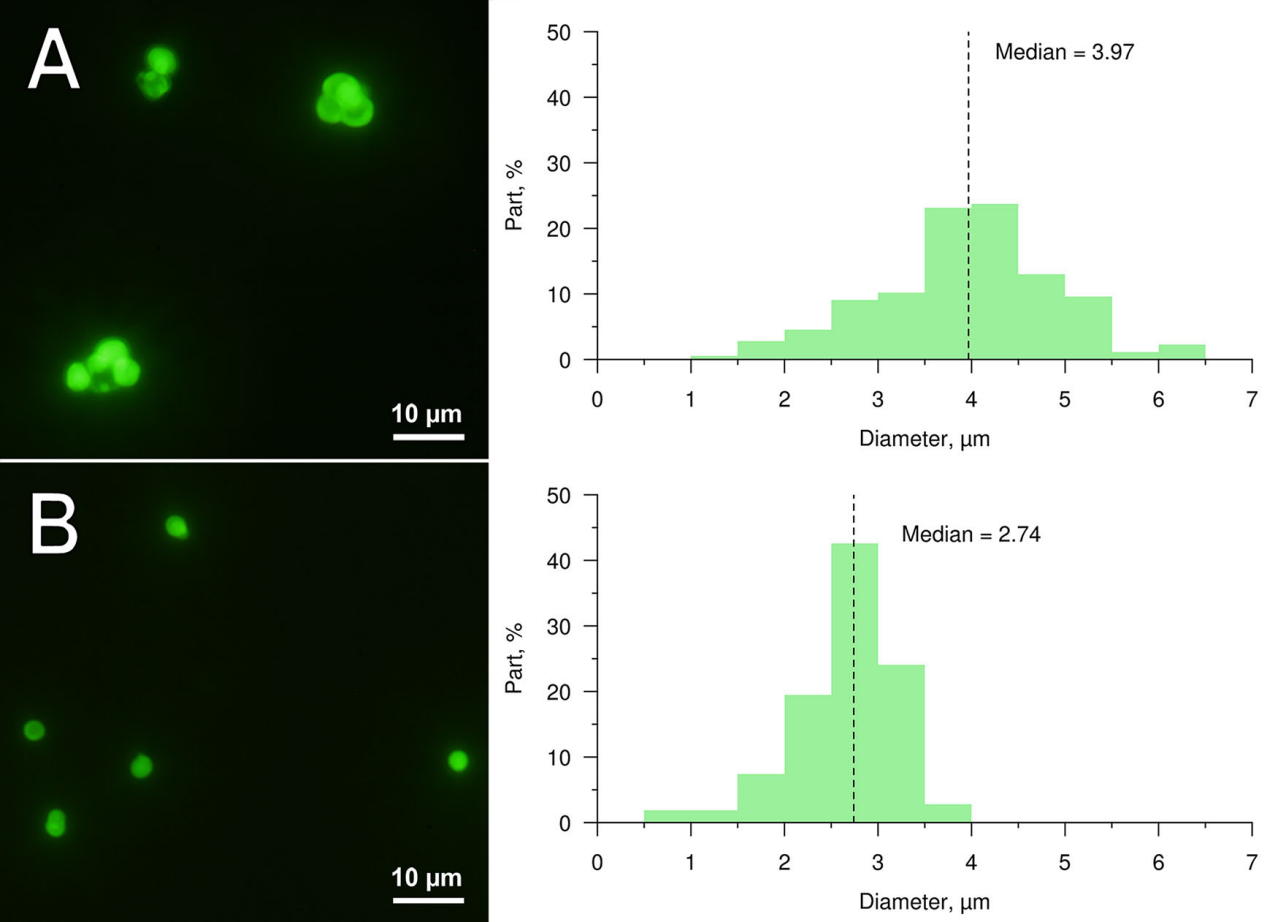
**Images and size profiles of prepared PMs-PEG containing FITC-BSA (used for investigation of distribution and stability).** Median diameters are approximately 4.0 µm (A) and 2.7 µm (B). Fluorescent images of PMs-PEG are obtained in green channel.

**Fig. 2.**
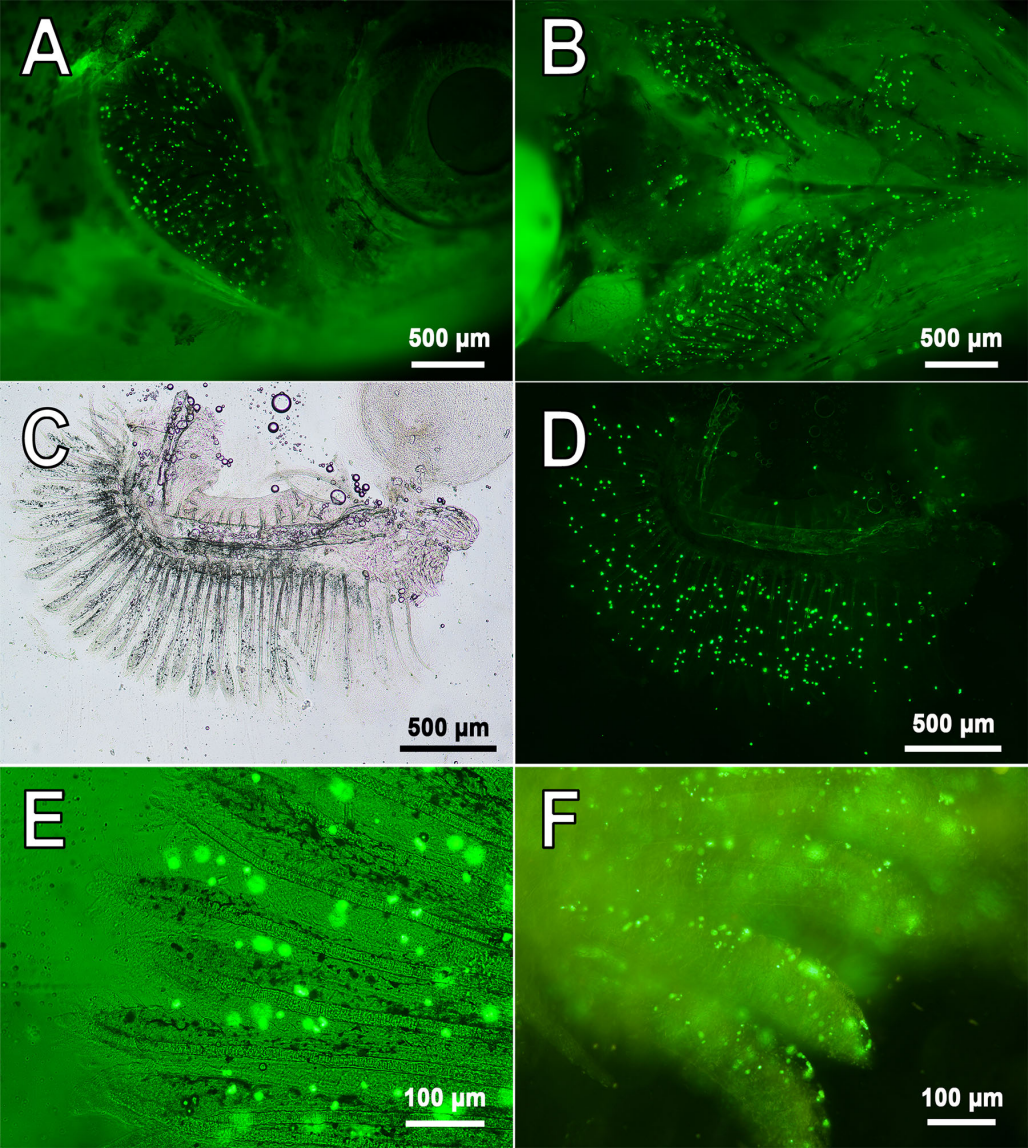
**Representative images of PMs-PEG in capillaries of zebrafish gills.** (A-E) 7 days following injection of PMs-PEG into the fish kidney. (F) 1 day following retro-orbital injection. A shows side view; B shows bottom view; C-F show isolated gill arches. Fluorescent images of PMs-PEG are obtained in green channel.

**Fig. 3.**
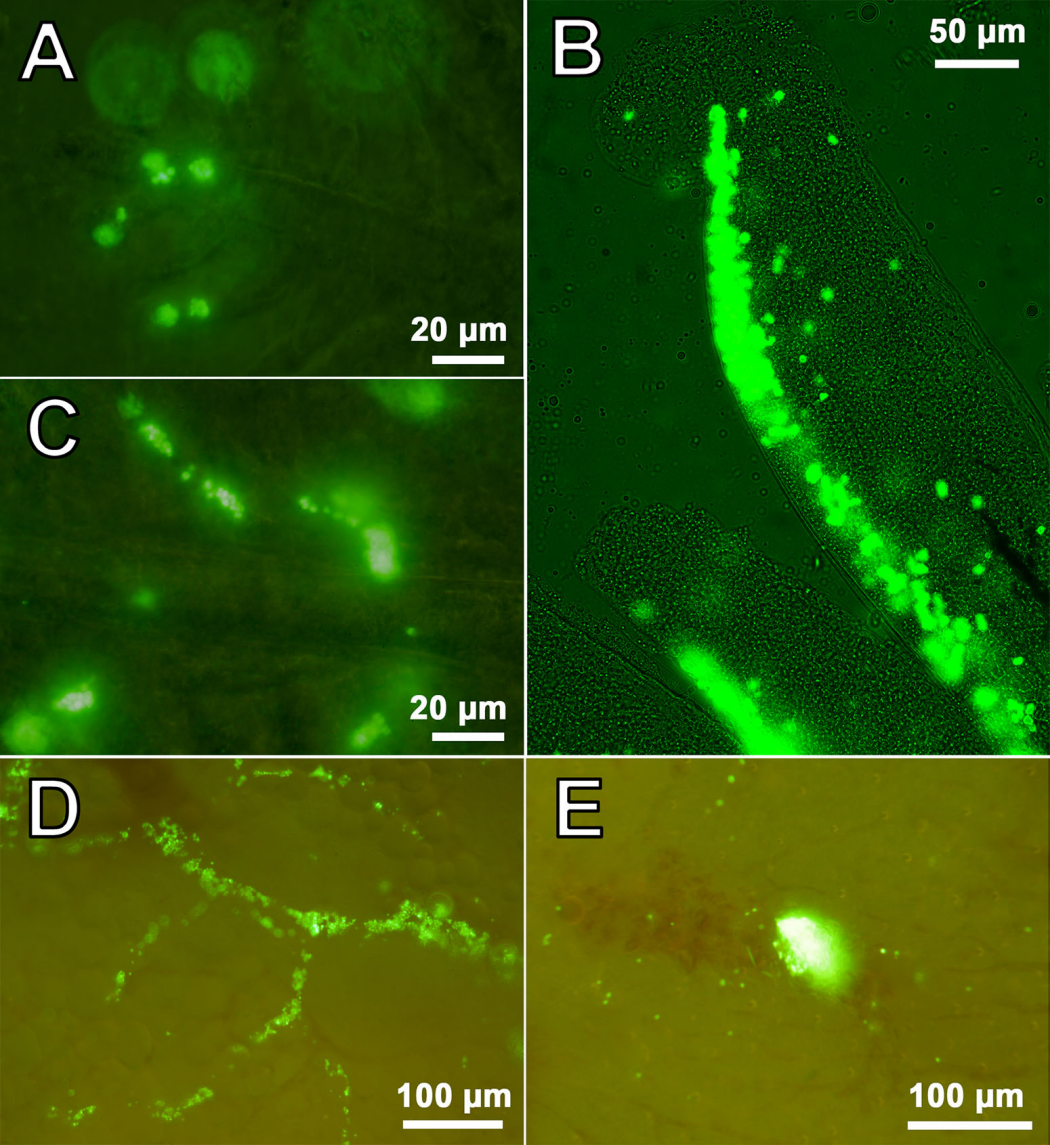
**Selected images of aggregates of PMs-PEG in zebrafish immediately after injection.** (A-C) Gill filaments of *D. rerio* following retro-orbital (A,C) or intrarenal (B) injection. (D,E) Aggregates of PMs-PEG in a hepatic vessel of some individuals of *D. rerio* following retro-orbital injection. Fluorescent images of PMs-PEG are obtained in green channel.

After delivery into the fish bloodstream (by injection into renal parenchyma or retro-orbital sinus), 3.9±0.9 µm (Fig. 1A) PMs-PEG in the bloodstream immediately spread throughout the body (Fig. 2; Figs S1-S4). Microscopy of dissected organs showed that within a few minutes after the injection, fluorescent PMs-PEG reached the heart, liver, gills, kidney, brain and other internal organs (Figs 2 and 3; Figs S1-S4). In the heart and big vessels, movement of free-floating PMs-PEG can be observed until the cardiac contraction goes on.

PMs-PEG were observed in vasculature for many days (Fig. 2; Figs S1-S4). Seven days following introduction of PMs-PEG, the highest concentrations of microcapsules (after both retro-orbital and intrarenal injections) were in the injection site and then in the gills and head kidney (Figs S1C-F, S2A-B and S3). PMs-PEG were relatively rare in the muscle tissue distant from the site of injection, in the gonads and in the fin capillaries. Microcapsules continued to be found in the fish heart one week after injection, indicating their free circulation (Fig. S2A,C).

Microscopy of the internal organs revealed sporadic aggregates of PMs-PEG in the capillaries of gills (*n*=3), liver (*n*=2) and brain (*n*=2) of some individuals (total *n*=9). Such aggregates have the potential to cause a blockage of the small capillaries. However, no obvious pathological patterns, such as organ color change or extensive necroses, were found by visual and microscopic inspection in these areas (Fig. 3; Fig. S4). A minor hemorrhage was associated with a cluster of PMs in the fish liver (*n*=1) (Fig. 3E).

### Distribution of PMs-PEG after intramuscular injection

Intramuscular injection was also used to implant PMs-PEG in adult fishes and investigate the fate and distribution of microcapsules. Two different sizes of PMs-PEG (median diameters 2.7 and 4.0 µm, Fig. 1) were used to compare the fates of smaller and larger PMs-PEG in the tissues.

**Fig. 4.**
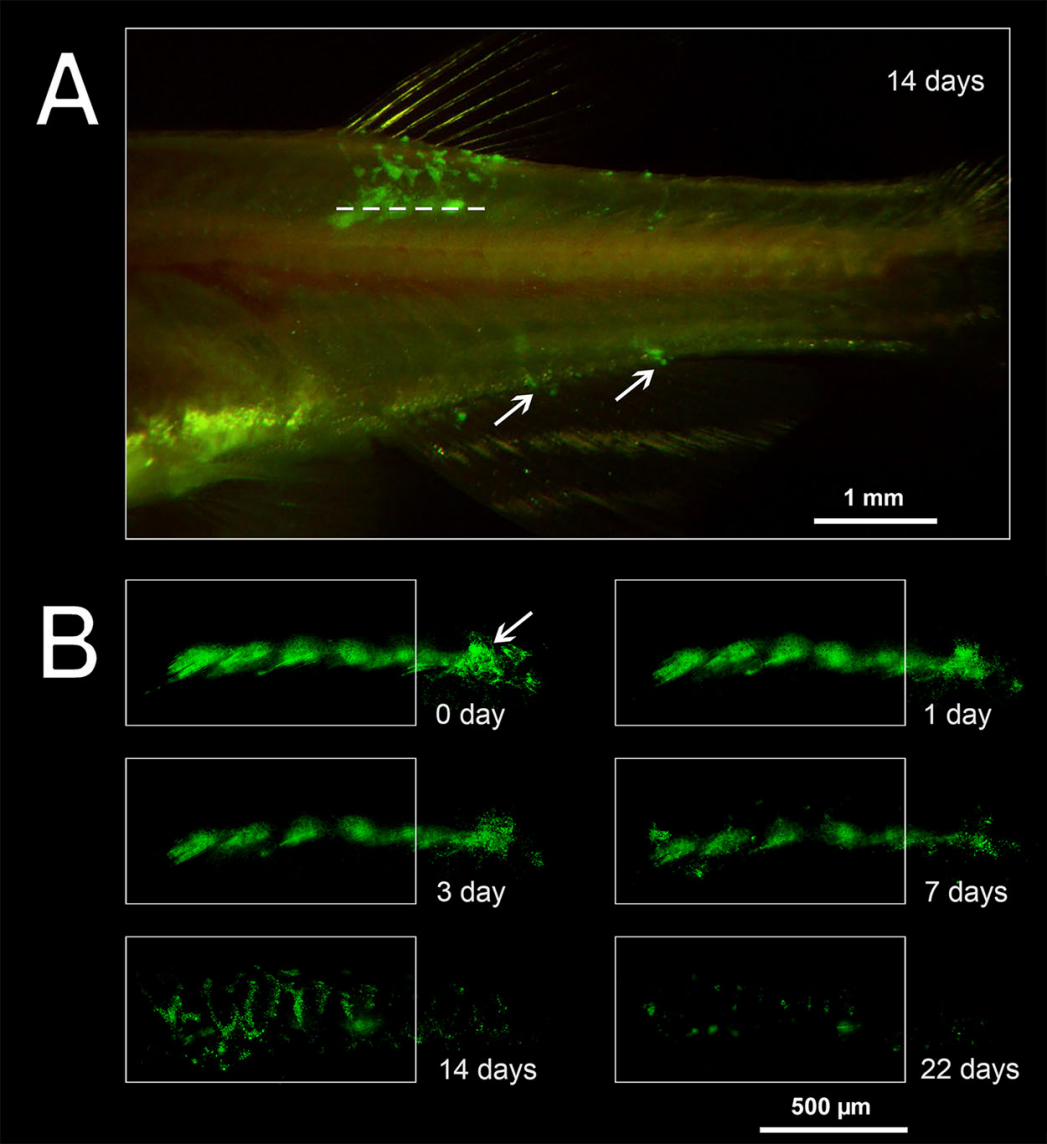
**Migration of PMs-PEG from the injection tract following intramuscular injection.** (A) Representative image of *D. rerio* 14 days following the intramuscular injection of PMs-PEG (dotted line corresponds to the injection tract). The arrows indicate fluorescence at the base of the anal fin rays. (B) Changes in the shape and area of FITC fluorescence in fish muscles. The frame indicates the analyzed region (see Fig. 5); the arrow marks the needle puncture inlet. Fluorescent images of PMs-PEG are obtained in green channel.

The fluorescence of intramuscularly injected PMs-PEG was clearly visible in the images as a bright luminous tunnel left by the needle across the segments of myotomes (Fig. 4). Microcapsules under the skin were increasingly blurred with depth. At the point of puncture inlet many bright dots were observed, and it was apparent that these were PMs-PEG spilt on the skin surface. Thus, to estimate stability of the fluorescence, we analyzed the region farthest from the injection inlet (Fig. 4B).

**Fig. 5.**
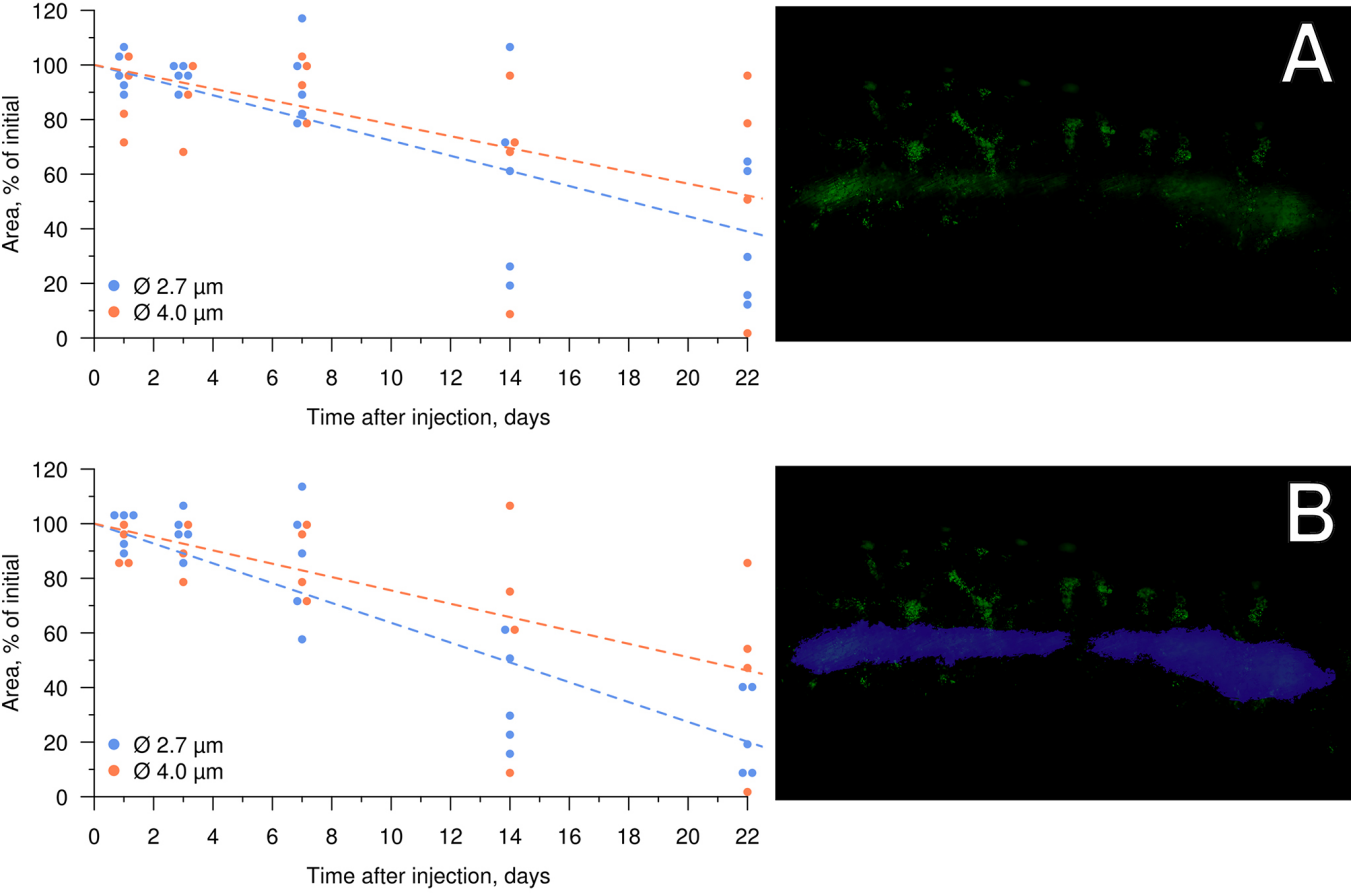
**Decrease of FITC fluorescence in fish muscles following injection of PMs-PEG with average diameters of 2.7 (*n*=5) and 4.0 µm (*n*=4).** (A) Total fluorescence. (B) Fluorescence in area of injection tract, which is designated by the purple selection on the right.

The area of fluorescence at the site of injection decreased with time (*n*=9), although the glow was detected at least three weeks post-injection. During the first week of observation, the area of fluorescence remained fairly constant in the region of injection (Fig. 5), with a decrease of approximately 12% in fishes injected with both 2.7 and 4.0 µm PMs-PEG. At 7 days after administration, migration of fluorescent dye by phagocytes was observed (see below), which appeared as clearly visible, separate fluorescent dots out of the injection tract. This fluorescence pattern was distributed along regular branched tracks, which were similar in appearance to the intersegmental spaces of myotomes. After 14 days, fluorescent objects were observed at the base of the dorsal fin rays and the base of the anal fin rays, which are on the opposite side of the fish body in the ventral direction from the injection site (Fig. 4A). Because the migrating elements spread out and increased the fluorescent area, the fluorescence of the migrating elements was subtracted in the analysis to assess the decreasing rate of fluorescence in the puncture tunnel only (Fig. 5B).

The size of PMs-PEG did not significantly affect the rates of decrease of fluorescence in muscles during the observation time. Fluorescent areas after the introduction of both types of PMs-PEG continued to decrease at a constant rate of approximately 15-25% per week. However, microcapsules with an average diameter of 2.7 µm tended to leave the injection site faster than the 3.9 µm PMs-PEG.

### Histological analysis of fish muscles after injection of PMs-PEG

**Fig. 6.**
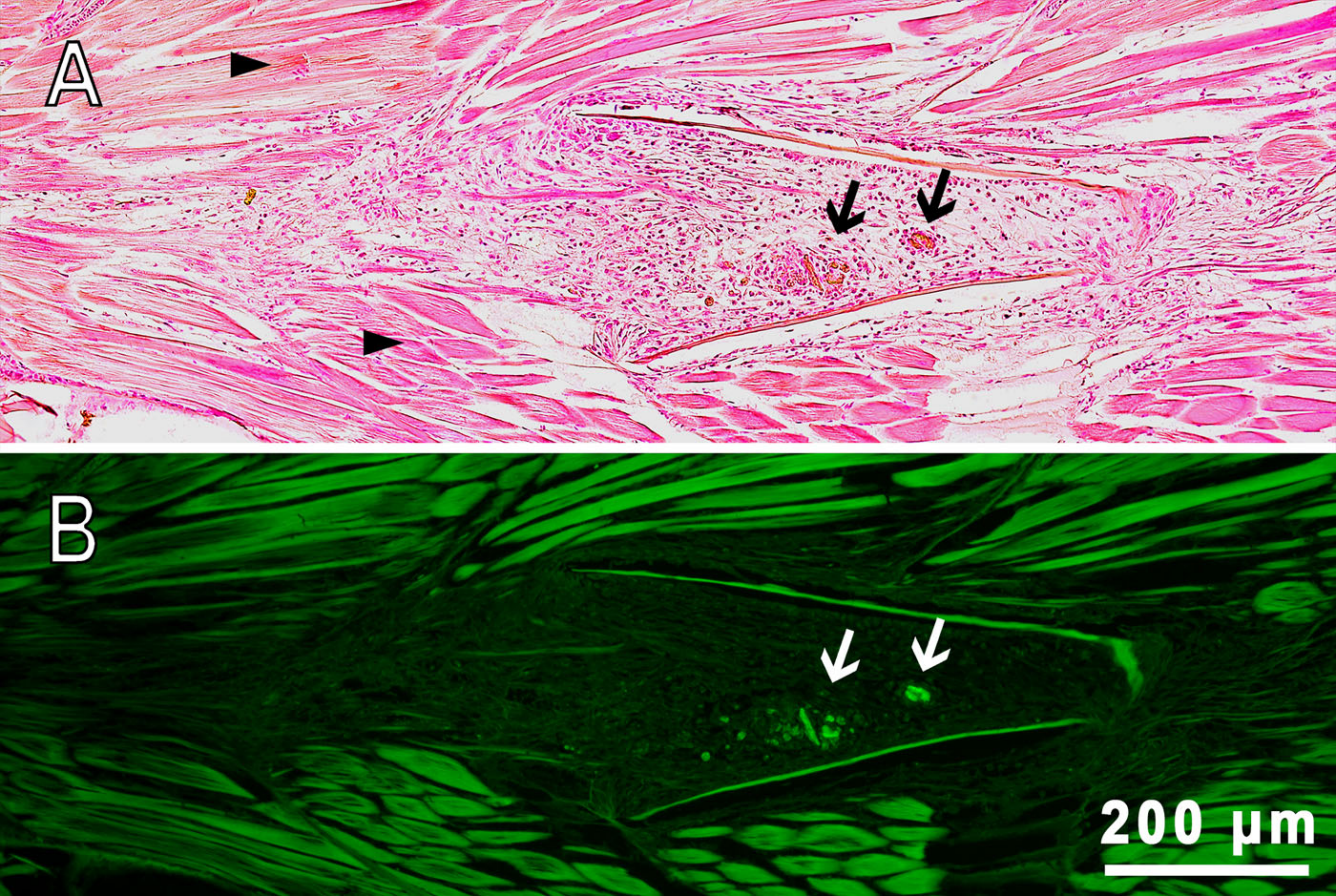
**Sagittal section of fish muscles of *D. rerio* 22 days following the intramuscular injection of PMs-PEG.** (A) Transmission image, H&E stain. (B) Fluorescence image. The arrows indicate clusters of PMs-PEG surrounded by macrophages (granulomas); arrowheads indicate normal muscle tissue composed of regular muscle fibers.

An injection tract was clearly distinguishable containing foci of FITC fluorescence on the serial histological sections of fish muscles. On day 22 after injection, the space of the hollow tract was filled with a relatively loose tissue, indicating the wound healing process was in the stage of granulation (Fig. 6). Granulation tissue is primarily composed of fibroblasts, which in the outer wound edge deposited collagen fibers and formed zones with dense, non-nuclear tissue (scar tissue). In more ‘recent’ areas of the wound, an ongoing process of chronic inflammation was occurring, characterized by the presence of young fibroblasts and a significant number of macrophages and lymphocytes removing mechanically damaged and necrotic tissues. In the inner zones of the wound, rounded structures consisting of macrophages tightly clustered around the aggregated fluorescent microcapsules were observed (Fig. S5). These structures were typical granulomas formed as a result of cellular immune response to the introduction of a foreign body. In some cases, macrophages that surrounded PMs-PEG fused together, organizing the so-called ‘multinucleated giant cells’ formation, which encapsulated the implant (Fig. S5E). The formation of giant cells with subsequent coating with a fibrin membrane is the final stage in the attempts of the immune system to eliminate an irritant, and occurs when the foreign body cannot be degraded and removed from the tissue.

**Fig. 7.**
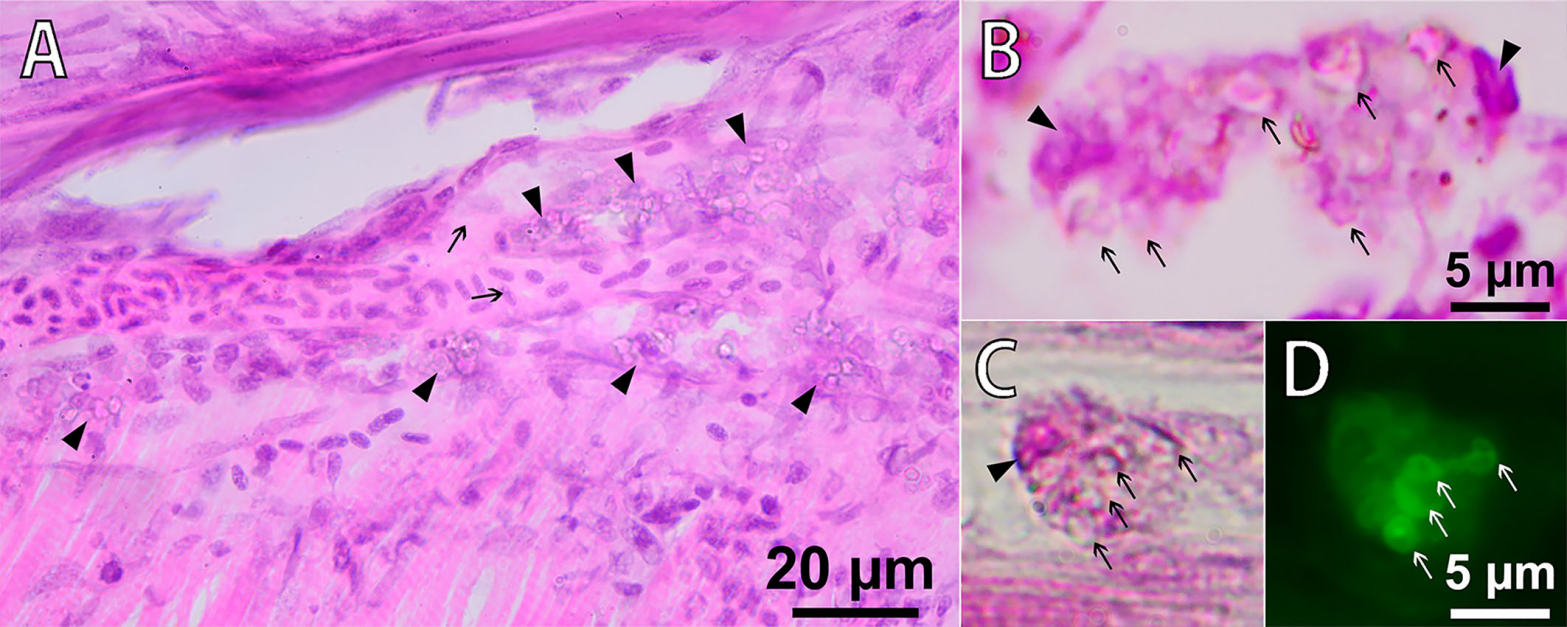
**Phagocytosis of PMs-PEG in fish muscles 22 days following the intramuscular injection of PMs-PEG.** (A) Microsection of *D. rerio* muscle, H&E stain. The arrows indicate a capillary filled with erythrocytes. The arrowheads indicate macrophages loaded with PMs-PEG (cells with granulated cytoplasm) migrating along the outer walls of blood capillaries in the lymphatic ducts. (B,C,D) Macrophages engulfing PMs-PEG. Note intact microcapsules (arrows) in the cytoplasm of phagocytes. The nuclei of the cells are marked with arrowheads. Fluorescence image (D) shows dye localization inside of intact microcapsules in the macrophage cytoplasm.

**Fig. 8.**
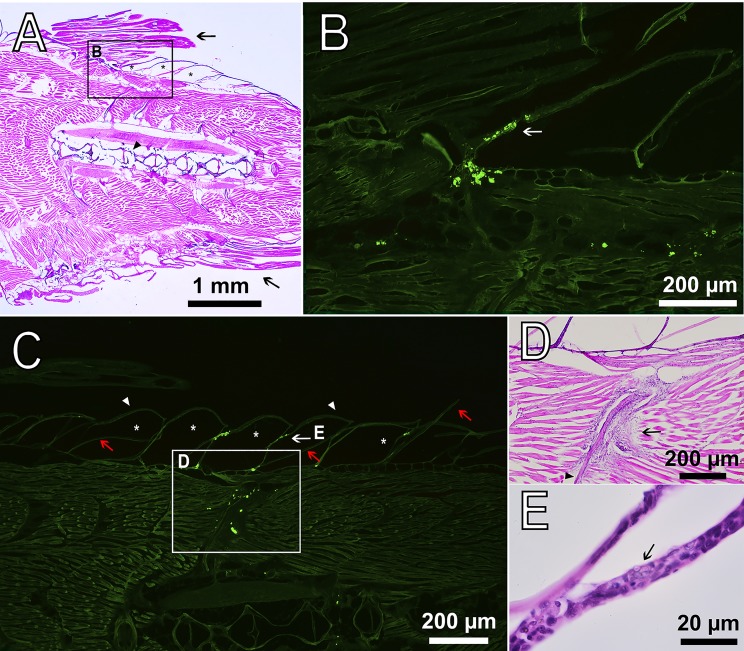
**Sagittal section of *D. rerio* 22 days after the intramuscular injection of PMs-PEG.** (A) Fish tail with dorsal (upper arrow) and anal fins (lower arrow), H&E stain. The asterisks indicate the scale pockets; the arrowhead indicates the backbone. (B) Fluorescence image scaled from A shows accumulation of macrophages loaded with PMs-PEG at the base of the dorsal fin. The arrow indicates the epidermis that covers the scales. Fluorescent images of PMs-PEG are obtained in green channel. (C) Fluorescence image of clusters of phagocytes loaded with PMs-PEG migrating from the muscle tissue toward the fish skin. The asterisks indicate the scale pockets; arrowheads indicate the epidermis; red arrows indicate the scale plates. (D) Figure scaled from C showing basophilic coloration around bone (arrowhead) indicating leucocyte infiltration and inflammation process, H&E stain. (E) Figure scaled from C shows loaded phagocyte (arrow) in the epidermis of scale, recognized by the granulated cytoplasm, H&E stain.

Migration of PMs-PEG outside the injection tract was observed along the outer walls of blood capillaries between the myomeres, apparently in lymphatic ducts (Fig. 7A). Despite lymph vessels of fishes having access to the primary blood system (Fischer and Steffensen, 2008; Rummer et al., 2014), we did not observe any microcapsules in capillaries of muscles. Free PMs-PEG were not observed and were found only as clusters inside phagocytic cells. Loaded phagocytes had typical bubble structures of micrometric size in their cytoplasm, easily detected by transmission imaging (Fig. 7). Fluorescent microscopy showed that fluorescence of the dye was localized inside these spherical structures, indicating that microcapsules did not lose their integrity in the phagocytes. Microphotographs showed that by day 22 after injection, loaded phagocytes migrated beneath the surface of the skin at the base of the fin rays and then continued to move into the epidermis covering the scales (Fig. 8).

## DISCUSSION

Some technical difficulties are associated with application of polyelectrolyte microcapsules loaded with fluorescent probes for *in vivo* sensing. The intensity and stability of the signal should be sufficient for reliable spectral detection, which require either sufficient concentration of PMs in the organism fluids or their accumulation in specific areas of interest. The spread of any particle in the body depends on the strategy of administration and the mobility, which is determined by the particle size, form and surface properties.

In this study, we tested PAH/PSS-based PMs covered with PLL-g-PEG [poly(L-lysine)-graft-poly(ethylene glycol) co-polymer] as carriers for a fluorescent indicator. The applied PMs-PEG were characterized by micrometric size and a flexible shell of nanometers thickness (Kreft et al., 2007). Particles of such caliber (more generally, from 0.5 to 100 µm) cannot pass the histo-hematic barrier (Yuan and Rigor, 2010). Therefore, being administered intravenously, PMs-PEG cannot leave the circulatory system. Similarly, when administered interstitially, PMs-PEG cannot be rapidly washed by blood from the site of injection. Therefore, microcapsules of this size loaded with an indicator probe are useful for continuous monitoring or visualization of the circulatory system (Yashchenok et al., 2016) and interstitial fluids. This caliber of particle can also be applied in practice for investigation of the lymphatic system (Andorko et al., 2014), vaccine formulations (De Geest et al., 2012), therapy of solid cancer (Kennedy, 2014; Shao et al., 2015) and ‘smart’ tattoo ink fabrications (Ruckh and Clark, 2014). However, some special techniques can be proposed to make PMs enter (or even pass) the endothelium of blood vessels, for example, by applying PMs with embedded magnetite nanoparticles under magnetic field (Voronin et al., 2017).

Previously, Sadovoy et al. (2012) administered 1-2 µm PMs-PEG in the pericardium of zebrafish embryos and reported that the manipulation did not cause any disorders of development, which confirmed the possibility of implanting the encapsulated fluorescent sensors into living organisms. One week after injection, PMs-PEG were registered in distant fish organs, including the brain, eyes, endoderm and the spinal cord; and thus, they spread in the circulatory system. The applied PLL-g-PEG cover was proposed to significantly improve the biocompatibility of the microcapsules in comparison with uncovered PMs and decrease the negative effects of microcapsule administration. Thus, the type of PMs-PEG has shown desirable properties for implantable sensors: stability of the shell (non-biodegradable) for long-term observations; shell flexibility facilitating PM traffic in tight capillaries; and high biocompatibility. However, in their study, the concentrations used were not provided, and the allowable concentrations that had no toxic or teratogenic effect on fish were not established.

To investigate toxic potential of PMs-PEG, we introduced a high concentration of microcapsules into adult *D. rerio*. Because of the tiny size of zebrafish, intravenous injection is traumatic; therefore, microcapsules were injected in the middle portion of the kidney, which is enriched with capillaries. After successful administration, PMs-PEG immediately appeared in the gill capillaries and were easily detected by intravital microscopy after removal of the gill cover (Fig. 2). Likewise, the fluorescence of PMs-PEG was easily registerable in this organ *in vivo* using a spectrometer coupled to a microscope as described in (Borvinskaya et al., 2017).

Concentrations of PMs-PEG up to 10^6^-10^7^ microcapsules/µl were used in the experiments, and these concentrations were apparently the maximum, because at higher suspension concentrations of PMs-PEG, the lumen of the injection needle began to clog. However, these concentrations were more than sufficient to introduce enough PMs-PEG for registration of spectral signal and physiological measurements. Moreover, the LD_50_ value for toxic effect was not reached during these experiments, confirming the low direct toxicity of PMs-PEG to the adult fish at the level of the organism. However, with the method of administration (injection in the fish kidney), only a part of the suspension directly entered into the bloodstream, with the excess spilling into the body cavity.

The highest death rate of approximately 10-15% was observed immediately after injection and was most likely associated with the trauma and stress caused by the procedure. The mortality then did not exceed that level in either the experimental or control group (received 0.9% NaCl solution). The type of encapsulated dye (i.e. FITC-BSA, FITC-Dextran or SNARF-1-Dextran) did not significantly affect fish mortality.

Micrometric components may pose a risk of obstruction in blood capillaries. Particles with limited mobility likely begin at the approximate size of blood cells, which in fishes ranges from 5 to 30 µm depending on the species (Gregory, 2003). Therefore, particles larger than 30 µm can potentially clog some capillaries and provoke thrombi and minor strokes. Because the PMs have soft flexible shells, their mobility is expected to be greater than that for solid particles of the same diameter. Similarly, the elastic membrane of red blood cells permits squeezing through tight capillaries (She et al., 2012). This feature is important for preventing blood clogs in small capillaries and is one of significant advantages of PMs. In our study, we observed that 3.9±0.9 μm PMs-PEG easily traveled through the zebrafish capillaries and, within the first minutes after injection, spread to the primary fish organs (gills, liver, kidney and brain) (Fig. 2; Figs S1-S4). However, mortality tended to be slightly higher in fishes injected with larger PMs-PEG and lower in fishes injected with microcapsules of smaller diameter (Table S1).

Microcapsules may form agglomerates with one another and with blood elements; thus, capillary occlusion most likely caused higher mortality of fishes injected with PMs-PEG at a concentration of approximately 4×10^6^/µl than fishes injected with lower concentrations (Table S1). Although most microcapsules in the fish organs were distributed one by one (Fig. 2; Figs S1-S4), single clusters of microcapsules that had the potential to cause a blockage of the small capillaries were found during the external examination of zebrafish organs (3 of 9 fishes) (Fig. 3). However, we did not observe marked blood congestion in these areas, which indicated that blood elements kept moving through the capillaries. Therefore, further histological study is required to determine whether ischemia symptoms occur in the regions in which such aggregates form.

Obtained results are consistent with a recent *in vivo* experiment investigating distribution of 3 μm PMs (with embedded magnetic nanoparticles) in the mesenteric vessels of rats (Voronin et al., 2017). PMs tended to aggregate in the bends and bifurcations in the network of microcapillaries due to blood turbulence slowing down the blood flow. Histological analysis showed that blood flow in big vessels remains intact, but PMs block some small mesenteric vessels. However, it should be noted that this study involved two factors facilitating the formation of aggregates: no special coverage was applied to reduce aggregation of PMs, and they were concentrated in the studied region by external magnetic field.

Retention in the network of branched and curved capillaries was shown in our study: over time, PMs-PEG tended to deposit in capillaries of organs with a rich blood supply (Fig. 2; Figs S2-S3). Therefore, kidney, gills, liver, spleen and brain are the organs that may be most affected by the introduction of large concentrations of microcapsules. The same results were obtained when microcapsules were injected into the tail vein of mouse (Yi et al., 2014; Shao et al., 2015). In those studies, accumulation of microcapsules was noted mainly in capillary-rich liver and spleen, as well as in the vascular network of tumors, which is a valuable property that allows the use of PMs for the visualization and treatment of solid cancers (Shao et al., 2015). The studies on mice do not report any obvious effects of PMs administered intravenously on morphology of main internal organs; however, special attention should be paid to assessment of chronic effects of microcapsules on these organs.

In contrast to systemic injection, microparticles administered interstitially have low mobility (Tang et al., 2009), and therefore, stable aggregates are more likely formed in the injection site. Only for the smallest size range (0.01-1 µm), some migration has been shown previously due to diffusion into the intercellular space (Molenda et al., 2008; Khetarpal et al., 2010; Facca et al., 2010). However, particles in the size range from 0.1 to 6 µm can be engulfed by phagocytic cells, which then transport the particles to the draining lymphatic nodes for presentation to T-cells (De Geest et al., 2006; Molenda et al., 2008; De Koker et al., 2007; Champion et al., 2008; Matsika et al., 2013; Cheung et al., 2014).

In this study, we observed distribution of the encapsulated fluorescent dye in fish tissues after intramuscular injection of PMs-PEG. Fluorescence of the dye was detected at least 3 weeks after injections, and therefore, during this time, the sensor signal was registerable (Fig. 4B). During the first week, the coloration was the most stable; however, a progressive decrease of the fluorescence occurred during the second and third weeks of the experiment, accompanied by migration of the fluorescent dye from the injection site. The decrease of fluorescence could be partly related to destruction of the microcapsules and leaching of the dye, but this effect was not investigated. However, a significant part of the fluorescence decrease was related to the migration of the dye out of the injection tract, which was caused by transport of microcapsules by phagocytic cells. The pattern of fluorescence distribution in fish muscles (Fig. 4) corresponded to the architecture of fish lymphatic vasculature (Isogai et al., 2009; Okuda et al., 2012). Histological analysis confirmed that fish immune cells recognized and attacked the introduced microcapsules. The PMs-PEG were transported from the injection site by phagocytic cells along the lymphatic ducts under the fish skin, and then to the epidermis (Figs 7 and 8). Fish skin mucosa is an inductive site containing a skin-associated lymphoid tissue (SALT) represented by secretory cells, lymphocytes, granulocytes, macrophages, and dendritic cells (Lugo-Villarino et al., 2010; Salinas et al., 2011; Xu et al., 2013). Presumably, phagocytes delivered the absorbed microcapsules to the secondary lymphoid tissue to present the antigen for activation of antigen-specific leukocytes.

Recognition of uncovered microcapsules by immune cells was shown earlier *in vitro*. De Koker and co-workers reported moderate tissue reaction to the subcutaneous administration of dextran sulfate/poly-L-arginine-based microcapsules (biodegradable). Within sixteen days, most of the microcapsules were engulfed by macrophages and destroyed (De Koker et al., 2007). Sulfate/poly-L-arginine microcapsules delivered in the pulmonary system to mice were internalized by macrophages and dendritic cells, and transported a long distance to the draining lymph nodes inside the body (De Koker et al., 2010). PMs composed of PSS and PAH are also engulfed by phagocytes, but unlike dextran-based microcapsules, these PMs retain their integrity at least 60 h after cellular uptake and even after incubation in pronase solution for several days (De Geest et al., 2006). Similar to De Geest and colleagues, we observed microcapsules filled with FITC in the cytoplasm of phagocytic cells, most likely macrophages (Lugo-Villarino et al., 2010). In our study, the PMs-PEG inside the phagocytes remained intact for at least 22 days after injection, supporting long-term non-biodegradability of the PAH/PSS/PLL-g-PEG shell.

Extent and rate of phagocytic clearance depend on the size and surface properties of PMs. The absorption maximum of macrophages occurs at a particle size of approximately 3 µm (Champion et al., 2008); thus, the caliber of PMs-PEG used in this study was presumably subjected to attack by leukocytes. Consistent with the maximum absorption of macrophages, the 2.7 µm median-sized PMs-PEG should have been more available for cellular uptake than those at 4.0 µm. The rate of fluorescence degradation in our experiment was slightly higher for microcapsules of this lower caliber, although the difference was not significant.

To mimic the microcapsules from phagocytes, the PEG coating of PMs was applied. PEGylation of PMs is typically used to prevent non-specific protein adsorption, opsonization, and subsequent cellular uptake (Poon et al., 2011; Morton et al., 2013; Cui et al., 2014). To immobilize PEG on charged polyelectrolyte microcapsules, copolymer polyethyleneglycole-grafted poly-L-lysine (PLL-g-PEG) was applied, as previously suggested (Heuberger et al., 2005; Sadovoy et al., 2012). Poly-L-lysine residues adsorb on PSS-terminated microcapsules through electrostatic interaction, with PEG chains stretched out perpendicularly to the surface. Wattendorf and coworkers (2008) demonstrated that PLL-g-PEG coatings on 1.2 and 4.8 µm microcapsules efficiently shielded them from cellular recognition, measured by a decline in phagocytosis of PMs by dendritic cells and macrophages up to 85–90%. Cellular internalization or association of PLL-g-PEG-coated microcapsules was minor during a 4 h experiment, in contrast to uncoated PMs or those coated with PLL only, which were readily incorporated. The PLL-g-PEG coating is stable for at least 4 weeks in buffer and human serum. No absorption of serum proteins was detected during storage, and the cellular recognition of incubated microcapsules by phagocytic cells was negligible (Wattendorf et al., 2008).

Immune response to PEGylated microcapsules can be caused for different reasons. Wattendorf and colleagues (2008) noted that even submicrometer-sized defects in the final PEG layer might lead to their recognition by phagocytes. Thus, any mechanical deformation of the microcapsule shell, insufficient PEG density on the surface or other characteristics that vary from batch-to-batch or within a batch in the fabrication of PMs-PEG may impair the biocompatibility. Additionally, PEG, despite poor opsonization, can nevertheless induce recognition by the immune system through complement activation (Knop et al., 2010). Therefore, other biomimetic non-biodegradable alternatives to PEG as terminal cover for PMs should be tested *in vivo* (Knop et al., 2010) to prolong the time of access of encapsulated sensors to intercellular space.

We observed apparent transport of microcapsules by phagocytic cells only on the second week after intramuscular injection (started between days 7 and 14). Such delay can be caused by late massive recognition of microcapsules due to the PEG coverage. We should mention that activation of immune cells' response can also be accelerated by the recruitment of phagocytes to remove debris of disrupted cells in the healing zone. Thus, we hypothesize that recognition by the immune system in a living organism could occur even slower following intravenous administration without wounding.

Therefore, according to our data, the prolonged biocompatibility of PMs-PEG reported previously (Wattendorf et al., 2008; Sadovoy et al., 2012) remains in question. Analysis of histological sections showed that on day 22 post-injection of PMs-PEG into the fish muscles only small fluorescent foci surrounded by phagocytic cells remained at the site of the injection, indicating a process of gradual resolution of microcapsule agglomerates (Fig. 6; Fig. S5). Most of the other space of the wound was filled with fibroblasts, regenerating blood vessels and fibrous tissue, which showed that the healing reaction developed according to a standard scenario. However, the specific morphological structures of granulomas and foreign-body giant cells indicated chronic inflammation processes provoked by PMs-PEG. Of note, the granuloma is an area of active chemical processes involving many proteases and oxidative enzymes, which must be considered when interpreting the signal of sensors in this formation.

### Conclusions

In the context of evaluation of physiological parameters *in vivo*, we administered PEG-covered polyelectrolyte microcapsules to adult zebrafish in this study to assess potential opportunities and risks related to application of these carriers for fluorescent sensors. The following two areas are the focus of discussion: the route of administration of PMs-PEG and the biological response to microcapsule insertion. These factors both significantly affect sensor signal recording and data interpretation.

The route of administration (interstitial or intravenous) determines the dynamic of distribution of microcapsules in the body. In this study, PMs-PEG 2-5 µm in diameter delivered into the bloodstream soon spread through the body and did not cause significant harmful effects. The administered amounts of PMs-PEG (up to 10^6^-10^7^ microcapsules/µl) were significantly below the LD_50_ dose but were absolutely sufficient for visualization and spectral signal recording for at least one week after injection. The PMs-PEG injected into muscles had a long half-life elimination period, which was similar for PMs with the median sizes of 2.7 and 4.0 µm. Phagocytes recognized microcapsules and actively transported them from the injection site; however, PMs-PEG retained their stability inside the phagocytes.

To summarize, the PMs-PEG showed negligible acute toxicity; however, chronic negative effects, such as induction of inflammation, require further consideration. The results obtained in this study are intended to facilitate further development and application of this technology for researchers in biological and medical studies.

## MATERIALS AND METHODS

### Preparation of PMs-PEG

Fluorescein isothiocyanate (FITC) conjugated with bovine serum albumin (FITC-BSA, FD20S; Sigma-Aldrich, USA) was used as the fluorescent label of PMs-PEG due to the intense fluorescence (Ex 494 nm, Em 512 nm) with high quantum yield (Sjöback et al., 1995). In the toxicity tests, two fluorescent dyes that conjugated with dextran, SNARF-1 (SNARF-1-Dextran, D3304; Thermo Fisher Scientific, USA) and rhodamine B isothiocyanate (RITC-Dextran, R9379; Sigma-Aldrich, USA), were also applied.

Hollow polyelectrolyte microcapsules filled with the fluorescent dyes were prepared using layer-by-layer assembly as previously described (Borvinskaya et al., 2017). The selected fluorescent dye was co-precipitated into cores of calcium carbonate by mixing 2 ml of a fluorescent dye solution with 0.615 ml of 1 M sodium carbonate and 0.615 ml of 1 M calcium chloride under stirring for 5 s, followed by washing 3 times in deionized water. The diameter of synthesized cores defines the size of prepared PMs-PEG, which varied by 1-2 µm from batch to batch for the same dye and also depending on the dye. The concentrations of the dyes used were 2.5 mg/ml for SNARF-1-Dextran, 2 mg/ml for FITC-Dextran and 1.2-2.5 mg/ml (depending on batch) for FITC-BSA. The prepared porous cores were then covered with 12 layers of oppositely charged polyelectrolytes: positive poly(allylamine hydrochloride) (PAH, 283215; Sigma-Aldrich, USA) and negative poly(sodium 4-styrenesulfonate) (PSS, 243051; Sigma-Aldrich, Belgium). Poly(L-lysine)-graft-poly(ethylene glycol) co-polymer (PLL-g-PEG, SZ34-67; SuSoS, Switzerland) was deposited as the final layer (Sadovoy et al., 2012). After dissolving the calcium carbonate cores in 0.1 M EDTA solution (pH 7.0), the structure of the PMs-PEG followed the formula (PAH/PSS)_6_/PLL-g-PEG with the selected fluorescent dye inside.

Concentrations of PMs-PEG and their diameters were evaluated using a counting chamber (hemocytometer). The images of PMs-PEG were obtained under a fluorescent microscope Mikmed-2 (LOMO, Russia) with an EOS 1200D camera (Canon, Japan) and analyzed using ImageJ 1.50d (NIH) (Schneider et al., 2012).

### Animals and housing

Adults of *Danio rerio* approximately 2.6 cm long and 0.3 g in weight were purchased in a local pet store and kept in polypropylene plastic tanks (1 fish per liter) filled with commercial bottled water from Lake Baikal. Fish were fed once a day with TetraMin commercial feed (Tetra Werke, Germany). Proper hydrochemistry was maintained by daily replacement of 20% of the water. Ammonium, nitrites and nitrates in the water were measured using semiquantitative commercial aquarium tests [Nilpa (Neva Tropic, Russia); Tetra (Tetra Werke, Germany)] and did not exceed 1, 0.3, and 12.5 mg/l, respectively. Water pH during experiments was 7.34-7.86, dissolved oxygen was maintained at 5-8 mg/l, and water temperature was approximately 19-20°С.

### Injections of PMs-PEG

PMs-PEG were delivered into the bloodstream of *D. rerio* by either intrarenal or retro-orbital injections, whereas intramuscular injections were performed to administer microcapsules interstitially. The work was conducted in accordance with the EU Directive 2010/63/EU for animal experiments and has been approved by the Animal Subjects Research Committee of Institute of Biology at Irkutsk State University. Before the injection procedure, each fish was individually immersed into 0.0002% methylene blue solution for 1.5 min to avoid the development of infections. After this treatment, fish were anesthetized for 1 min in clove oil suspended in water (0.1 ml/l), weighed and immobilized on a wet sponge (Cheung et al., 2014). Suspensions with different concentrations of PMs-PEG in sterile 0.9% NaCl solution were injected using a 31 G needle (Ø 0.25 mm) connected to a microinjector IM-9B (Narishige, Japan).

To introduce PMs-PEG into bloodstream, injections into a central bulge of the fish trunk kidney were performed in the middle of abdomen immediately under the lateral line. During the injection, the needle mechanically damaged renal parenchyma that is rich in capillaries and blood vessels, which allowed PMs-PEG entrance into the circulatory system. The introduction of PMs by retro-orbital injection was the second method of delivery into the fish bloodstream (Pugach et al., 2009). However, because this technique did not show satisfactory effectiveness during pretests, retro-orbital injection was not used further for PMs-PEG toxicity testing.

Treated fishes were rinsed with water, and then with surgical scissors, one operculum was excised. The success of the PMs-PEG delivery into the bloodstream was monitored by rapid inspection of denuded gills for fluorescent particles. In case of a successful injection, fluorescent PMs-PEG appeared immediately in the gills (see e.g. Fig. 2). For recovery, fishes were placed in tanks with aeration. The entire procedure required an average of 3-5 min per one fish.

Intramuscular injections were performed by puncture in the dorsal part of the tail of *D. rerio*. The needle was introduced at approximately 5 mm in the cranial direction along the dorsal fin, approximately 0.1-0.5 mm beneath the skin. After the procedure, fishes were rinsed with water to remove spilled suspension of PMs-PEG (if any), imaged and returned into their holding tanks for recovery.

### Organism-level toxicity examination

To evaluate acute and chronic effects of PMs-PEG administration, fishes were injected in the kidney with suspensions (1.6-2 µl) of various concentrations of PMs-PEG (4×10^3^-6×10^6^) of various sizes (average Ø 2.0-5.1 µl) and filled with several fluorescent dyes (Table S1). After the procedure, fishes were placed in a holding aquarium and monitored for up to 22 days. Deaths of fishes were recorded after the injection (when the fish did not recover within 30 min after anesthesia) and then once a day. In the controls of the experiment, fish received a 0.9% solution of NaCl instead of PMs-PEG suspension.

### Investigation of PMs-PEG distribution in the bloodstream

To study tissue distribution of PMs-PEG through the circulatory system, six individuals of *D. rerio* were injected into the kidney and three individuals received retro-orbital injections (Pugach et al., 2009). The injections were 1.6 µl of suspension of 7×10^5^ PMs-PEG per µl with a size of 3.9±0.9 µm (median=3.97 µm) and containing FITC-BSA (Fig. 1A). Three individuals (two injected into the kidney and one retro-orbitally) were sacrificed immediately after injection (0 day) and then at days 3 and 7 by immersion in 0.5 ml/l clove oil-water suspension for 10 min. The general inspection and imaging of fish were performed using a BX53 fluorescent microscope (Olympus, Japan) with D7100 camera (Nikon, Japan) or an SPM0880 zoom stereomicroscope (Altami, Russia) with U3CMOS05100KPA camera (Altami, Russia) and a blue laser (445 nm) coupled with optical fiber OV-KS0600-08 (Altami, Russia) for fluorescence excitation. Then, fish organs (gills, heart, liver, kidney and brain) were dissected (Gupta and Mullins, 2010), washed in saline, transferred onto glass slides, covered by glass coverslips and viewed under a Mikmed-2 fluorescent microscope (LOMO, Russia) with 1200D camera (Canon, Japan). Representative photos, if necessary, were automatically stacked or stitched in a panoramas using Adobe Photoshop CC software.

### Investigation of PMs-PEG distribution and stability after intramuscular injection

Two sizes of PMs-PEG, 3.9±0.9 µm (median=3.97 µm) and 2.7±0.6 µm (median=2.74 µm), were used for intramuscular injections (Fig. 1A and B, respectively). Concentration of PMs-PEG was adjusted for the fish from both groups (*n*=4 and 5) to receive approximately the same amount of dye (0.01 µg of dye per 1 μl of PMs-PEG suspension).

To determine the stability of fluorescence in the site of injection, all individuals were photographed immediately after the manipulation and on days 1, 3, 7, 14 and 22 post-injection. Before each examination, fish were anesthetized in a water suspension of clove oil (0.1 ml/l for 1 min). All photographs were taken using a 1200D camera (Canon, Japan) connected to a Mikmed-2 fluorescent microscope (LOMO, Russia) with identical camera settings and magnification. The digital photos were merged and stacked automatically using Adobe Photoshop CC software. Selection of a colored region and calculation of the area (number of saturated pixels in the green channel) were conducted in the same program using the built-in measurement tools by unified procedure.

To prepare Fig. 5, an additional package *beeswarm* (Eklund, 2016) for R (R Core Team, 2017) was used. The linear regression was built with the intercept forced to equal 100. The difference in rates of fluorescence degradation between PMs-PEG of different sizes was verified at each time point using the Mann–Whitney *U*-test with correction for multiple comparisons according to Hommel in R (R Core Team, 2017).

### Histological examination

For histological analysis, individual zebrafish were sacrificed 22 days after intramuscular injection in 0.5 ml/l clove oil-water suspension for 10 min. After removal of head and tail, fish bodies were dissected in half transversely and fixed in Bouin's solution (Sigma-Aldrich) at 25°C for 1 day. On the next day, samples were rinsed in tap water for one hour and stored in 10% neutral buffered formaldehyde. Biological specimens were embedded in paraffin routinely using an STP 120 Spin Tissue Processor (Thermo Fisher Scientific, USA) according to the protocol for fish tissues (Mikodina et al., 2009). Paraffin molds were sectioned at 6 μm in thickness by an HM-440 microtome (Thermo Fisher Scientific, USA). Tissue slides were first deparaffinized, then inspected by fluorescent microscopy using an Olympus CX61 microscope to detect PMs-PEG (since eosin has fluorescence spectra similar to that of FITC). The localization of the detected PMs-PEG was verified in series of adjacent sections for all samples to avoid possible misinterpretation of artifacts caused by the displacement of particles during cutting. Finally sections were stained with hematoxylin and eosin by standard procedure. Stained sections were studied under a light microscope and photographed with a D7100 camera (Nikon, Japan) connected to the microscope.

## Supplementary Material

Supplementary information
