# Supplementary material for: Distribution of PEG-coated hollow polyelectrolyte microcapsules after introduction into the circulatory system and muscles of zebrafish
Source: Biol Open. 2018 Jan 15;7(1):bio030015. doi: 10.1242/bio.030015 (PMC5829502; doi:10.1242/bio.030015)
Supplement: Supplementary information [file biolopen-7-030015-s1.pdf]

Biology Open (2018): doi:10.1242/bio.030015: Supplementary information

Supplementary information to

Distribution of PEG-coated hollow polyelectrolyte microcapsules after introduction into the circulatory system and muscles of zebrafish

Ekaterina Borvinskaya, Anton Gurkov, Ekaterina Shchapova, Boris Baduev, Igor Meglinski, Maxim Timofeyev

Table S1. Mortality of *D. rerio* after administration of PMs-PEG.

Encapsulated fluorescent dye	Average diameter of PMs-PEG, µm	Injection volume, µl	Concentration, microcapsules per µl	Mortality after injection, % (number of dead fish/number of fish)							
				1 h	1 d	3 d	6 d	7 d	9 d	14 d	22 d
Injection into trunk kidney											
SNARF-1-Dextran	5.1	2	$6\cdot10^5$	10% (2/20)	17% (3/18)	-	-	-	-	-	-
RITC-Dextran	2.0	1	$6\cdot10^6$	0% (0/20)	5% (1/20)	0% (0/15)	0% (0/10)	0% (0/10)	0% (0/5)	0% (0/5)	-
FITC-BSA	2.7	1.6	$4\cdot10^6$	5% (3/58)	4% (2/53)	2% (1/46)	3% (1/40)	4% (1/23)	0% (0/16)	0% (0/10)	0% (0/5)
FITC-BSA	2.7	1.6	$4\cdot10^5$	0% (0/7)	0% (0/7)	0% (0/7)	0% (0/7)	0% (0/7)	0% (0/7)	-	-
FITC-BSA	2.7	1.6	$4\cdot10^4$	0% (0/7)	0% (0/7)	0% (0/7)	0% (0/7)	0% (0/7)	0% (0/7)	-	-
FITC-BSA	2.7	1.6	$4\cdot10^3$	14% (1/7)	0% (0/6)	0% (0/6)	0% (0/6)	0% (0/6)	0% (0/6)	-	-
0.9% NaCl	-	1.6	0	15% (2/13)	0% (0/11)	0% (0/11)	0% (0/11)	0% (0/11)	0% (0/11)	0% (0/5)	0% (0/5)
0.9% NaCl	-	1	0	0% (0/24)	0% (0/20)	0% (0/15)	0% (0/10)	0% (0/10)	0% (0/3)	0% (0/3)	-
Injection into muscle											
SNARF-1-Dextran	5.1	1	$1.3\cdot10^3$	10% 2/20	10% 2/20	0% 0/10	0% 0/10	0% 0/10	-	-	-
SNARF-1-Dextran	5.1	1	$6.4\cdot10^3$	0% 0/20	0% 0/20	0% 0/10	0% 0/10	0% 0/10	-	-	-
SNARF-1-Dextran	5.1	1	$3.2\cdot10^4$	0% 0/20	0% 0/20	0% 0/10	0% 0/10	0% 0/10	-	-	-

Biology Open • Supplementary information

SNARF-1-Dextran	5.1	1	$1.6 \cdot 10^5$	0%<br>0/20	0%<br>0/20	0%<br>0/10	0%<br>0/10	0%<br>0/10	-	-	-
RITC-Dextran	2.0	1	$5.5 \cdot 10^6$	0%<br>0/21	5%<br>1/21	0%<br>0/15	0%<br>0/10	0%<br>0/10	0%<br>0/5	0%<br>0/5	-
0.9% NaCl	-	1	0	5%<br>2/40	3%<br>1/39	0%<br>0/25	0%<br>0/20	0%<br>0/20	0%<br>0/5	0%<br>0/5	-
Animal welfare											
No treatment	-	-	0	0%<br>0/34	0%<br>0/34	0%<br>0/22	0%<br>0/20	0%<br>0/20	0%<br>0/10	0%<br>0/10	-

Biology Open • Supplementary information

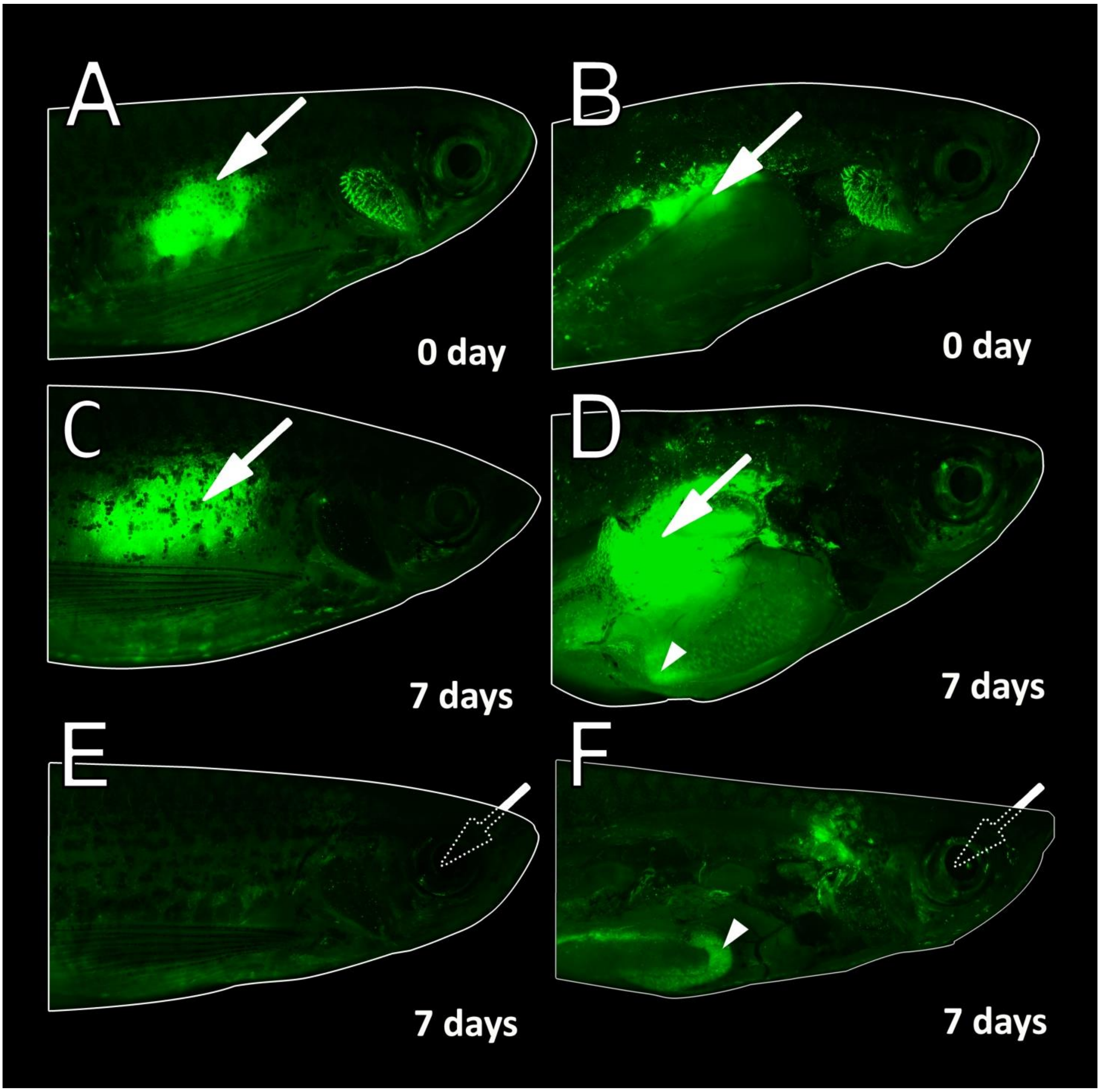

Fig. S1. Representative images of *D. rerio* following injection of PMs-PEG into the trunk kidney (A-D) and retro-orbitally (E-F). Fishes were photographed after gill cover was removed (A, C, E) and body wall was removed (B, D, F). Puncture sites are indicated with arrows. Note autofluorescence of intestine (arrowheads).

Biology Open • Supplementary information

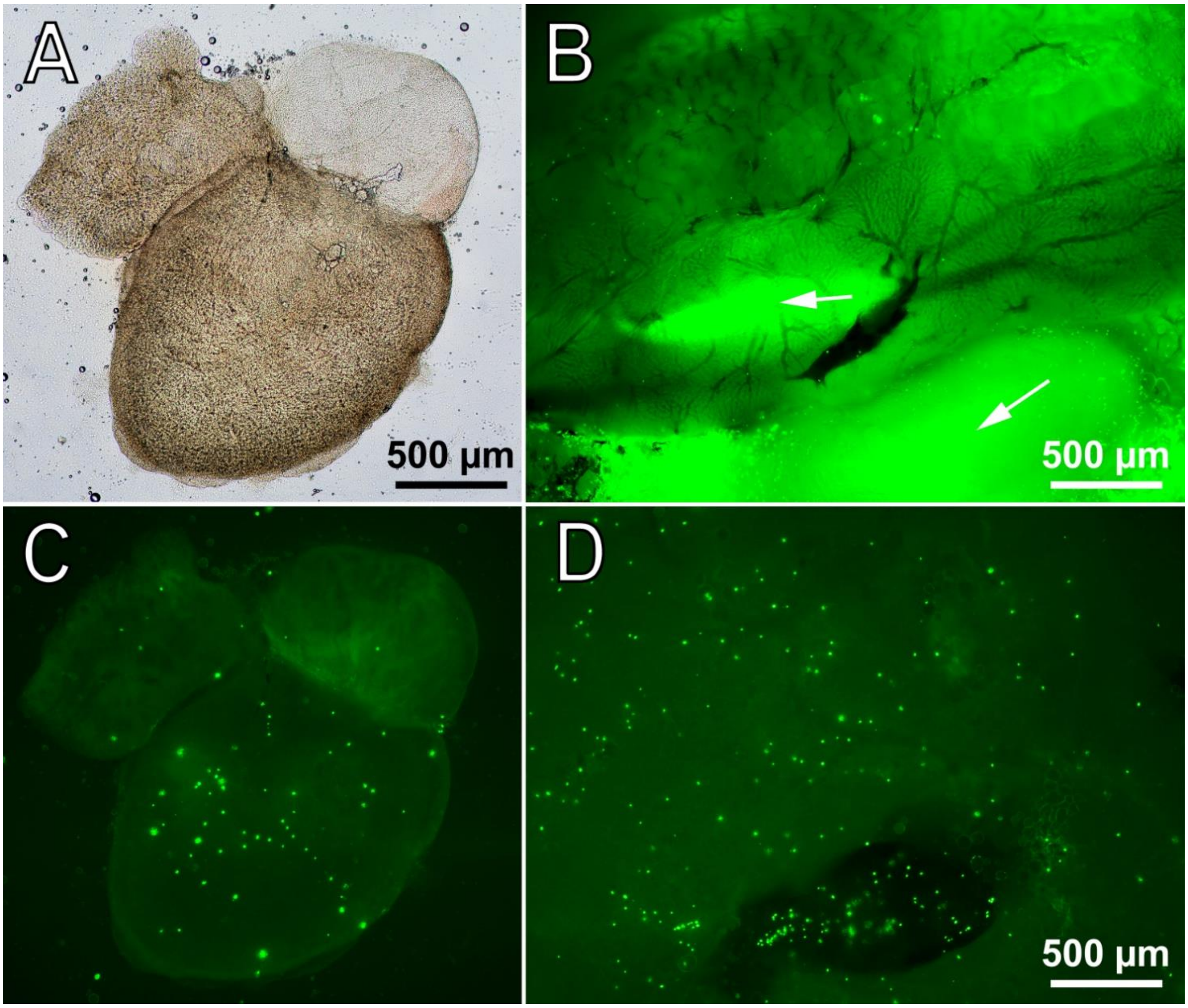

Fig. S2. Organs of *D. rerio* 7 days following PMs-PEG injection into the fish kidney. A — transmission image of fish heart. C — respective fluorescence image of fish heart. B, D — fish liver. Note autofluorescence of gallbladder (arrows).

Biology Open • Supplementary information

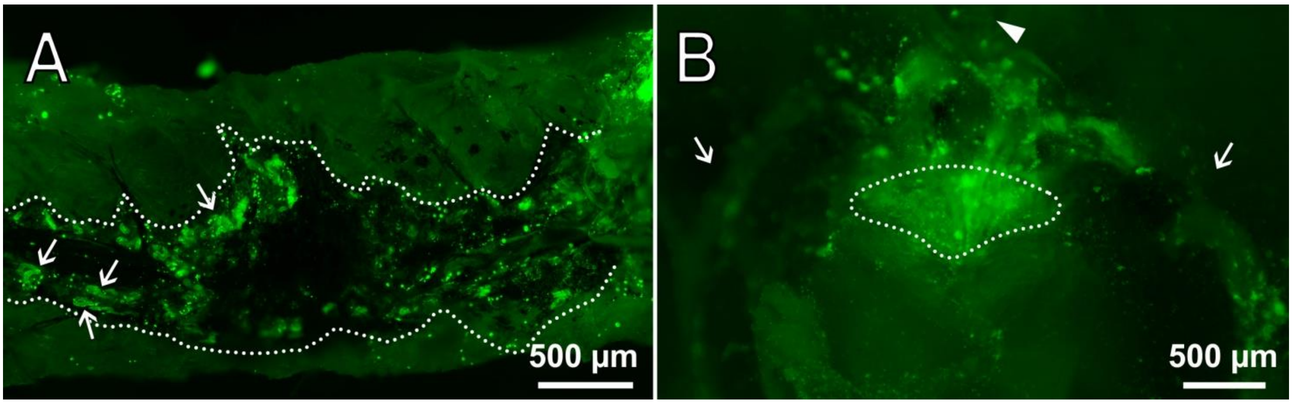

Fig. S3. Kidney of *D. rerio* 7 days following PMs-PEG injection. A — frontal plane of fish body (body wall removed) 7 days following PMs-PEG injection into fish kidney (dotted). Note autofluorescence of renal tubules (convoluted tubules marked by arrows). B — transverse section of *D. rerio* 7 days following retro-orbital injection of microcapsules. PMs-PEG accumulation in the fish head kidney (dotted). The arrows indicate ribs; arrowhead indicates the backbone.

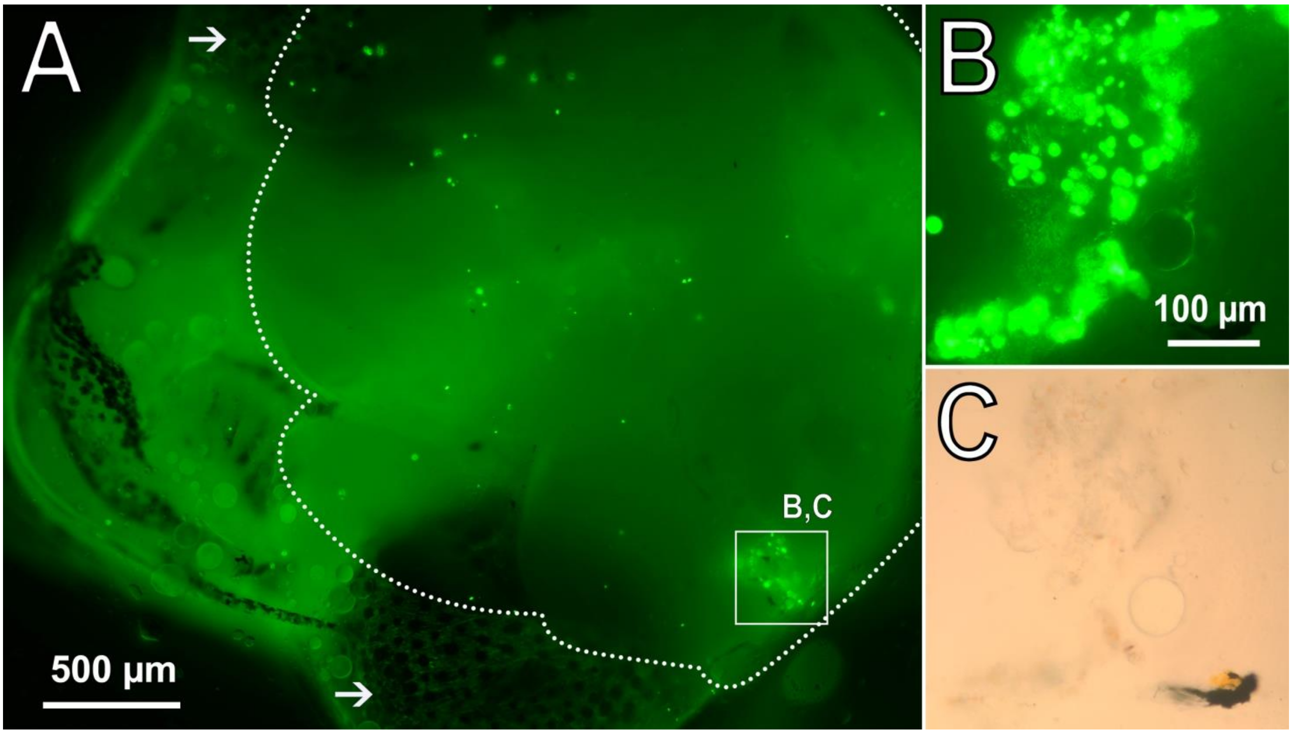

Fig. S4. The frontal plane of *D. rerio* head with removed bones of the cranial vault 7 days following PMs-PEG injection into the fish kidney. A — brain (flattened by coverslip, dotted) with fluorescent microcapsules. The arrows indicate fish eyes. B, C — figures scaled from S4A show agglomerate of PMs-PEG in fish brain detected by fluorescence (B) and transmission imaging (C).

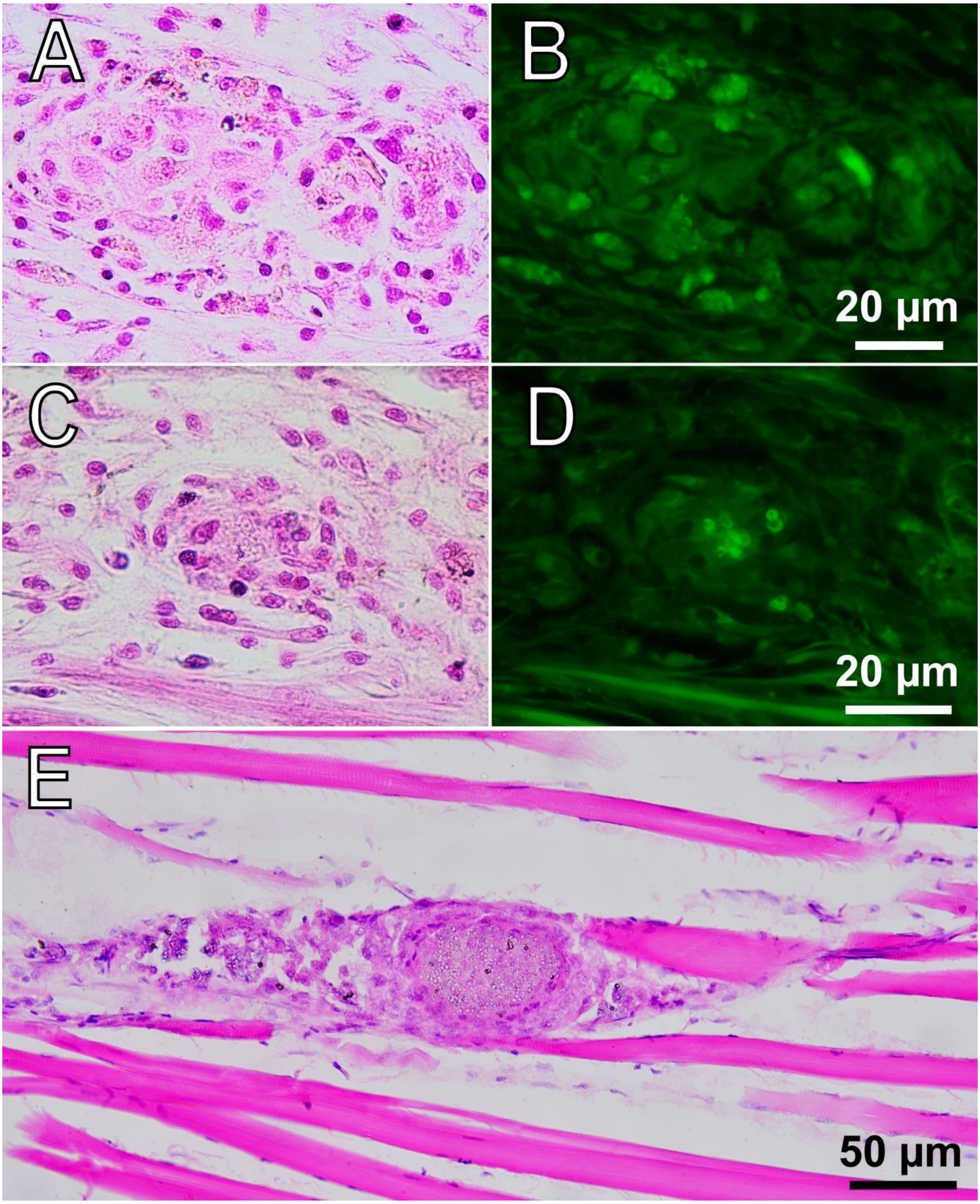

Fig. S5. Granulomas (A–D) and foreign body giant cell formation (E) in fish muscles 22 days following the intramuscular injection of PMs-PEG. A, C, E — transmission images, H&E stain. B, D — fluorescence images.
